# 2-Isopropyl-2-(6-meth­oxy-1,3-benzo­thia­zol-2-yl)-5,5-dimethyl-1,3-thia­zolidin-4-one

**DOI:** 10.1107/S1600536813019521

**Published:** 2013-08-07

**Authors:** Hendryk Würfel, Helmar Görls, Dieter Weiss, Rainer Beckert

**Affiliations:** a Institut für Organische Chemie und Makromolekulare Chemie, Universität Jena, Humboldtstrasse 10, 07743 Jena, Germany; b Institut für Anorganische und Analytische Chemie, Friedrich-Schiller-Universität Jena, Humboldtstrasse 8, 07743 Jena, Germany

## Abstract

The title compound, C_16_H_20_N_2_O_2_S_2_, crystallizes with two enanti­omers (*A* and *B*) in the asymmetric unit. The most noticeable difference between these two mol­ecules is the relative orientation of the benzo­thia­zole rings, with S—C—C—S torsion angles of −19.4 (2) (mol­ecule *A*) and 100.6 (1)° (mol­ecule *B*). The amide structure of the thia­zolidinone rings leads to inter­molecular hydrogen-bonded dimers of the *R* and *S* enanti­omers.

## Related literature

For chemi- and bioluminescence of firefly luciferin and related compounds, see: Jung *et al.* (1975 ▶); Naumov *et al.* (2009 ▶); White *et al.* (1979 ▶); Branchini *et al.* (2002 ▶). For structure modifications of firefly luciferin, see: Meroni *et al.* (2009 ▶); McCutcheon *et al.* (2012 ▶); Branchini *et al.* (2012 ▶); Würfel (2012 ▶). Luciferin and related structures are widely used in clinical and biochemical applications, see: Schäffer (1987 ▶); Kricka (1988 ▶); Josel *et al.* (1994 ▶); Shinde *et al.* (2006 ▶). All solvents were purified and dried according to Armarego & Chai (2009 ▶).
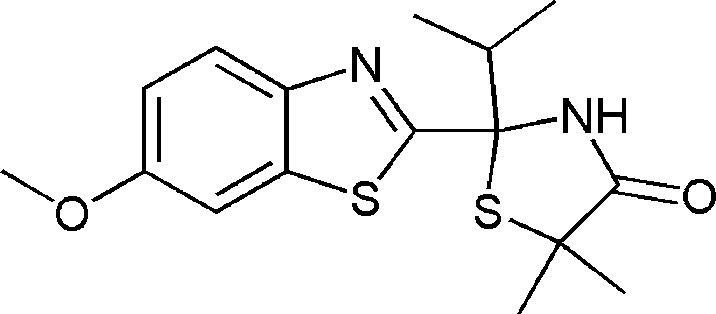


## Experimental

### 

#### Crystal data

C_16_H_20_N_2_O_2_S_2_
*M* *_r_* = 336.46
Triclinic, 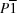

*a* = 11.3755 (3) Å
*b* = 11.9028 (3) Å
*c* = 12.5261 (3) Å
α = 86.122 (1)°
β = 85.949 (1)°
γ = 89.206 (1)°
*V* = 1687.86 (7) Å^3^
*Z* = 4
Mo *K*α radiation
μ = 0.32 mm^−1^
*T* = 133 K
0.06 × 0.05 × 0.05 mm

#### Data collection

Nonius KappaCCD diffractometer
10948 measured reflections
7580 independent reflections
6827 reflections with *I* > 2σ(*I*)
*R*_int_ = 0.019

#### Refinement

*R*[*F* ^2^ > 2σ(*F* ^2^)] = 0.039
*wR*(*F* ^2^) = 0.088
*S* = 1.08
7580 reflections
557 parameters
All H-atom parameters refined
Δρ_max_ = 0.37 e Å^−3^
Δρ_min_ = −0.28 e Å^−3^

### 

Data collection: *COLLECT* (Nonius, 1998 ▶); cell refinement: *DENZO* (Otwinowski & Minor, 1997 ▶); data reduction: *DENZO*; program(s) used to solve structure: *SHELXS97* (Sheldrick, 2008 ▶); program(s) used to refine structure: *SHELXL97* (Sheldrick, 2008 ▶); molecular graphics: *SHELXTL/PC* (Sheldrick, 2008 ▶); software used to prepare material for publication: *SHELXL97*.

## Supplementary Material

Crystal structure: contains datablock(s) I, global. DOI: 10.1107/S1600536813019521/fk2073sup1.cif

Structure factors: contains datablock(s) I. DOI: 10.1107/S1600536813019521/fk2073Isup2.hkl

Supplementary material file. DOI: 10.1107/S1600536813019521/fk2073Isup3.cml

Additional supplementary materials:  crystallographic information; 3D view; checkCIF report

## Figures and Tables

**Table 1:** Hydrogen-bond geometry (Å, °)

*D*—H⋯*A*	*D*—H	H⋯*A*	*D*⋯*A*	*D*—H⋯*A*
N2*A*—H1*NA*⋯O2*B*	0.81 (3)	2.15 (3)	2.9429 (19)	168 (2)
N2*B*—H1*NB*⋯O2*A*	0.77 (2)	2.04 (2)	2.802 (2)	173 (2)

**Fig. 1.**
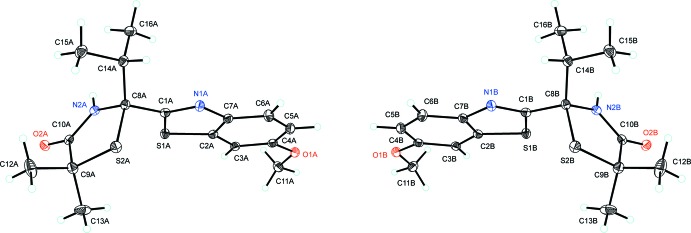
Molecular structure of the title compound with symmetry-independent molecules A and B; anisotropic displacement ellipsoids are shown at the 40% probability level.

**Fig. 2.**
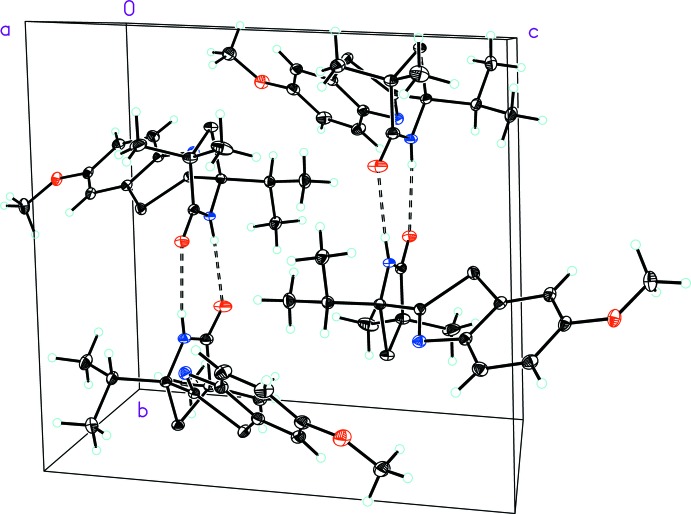
Crystal packing, viewed along a axis, showing hydrogen bonding between molecules A and B drawn as dotted lines.

**Fig. 3.**
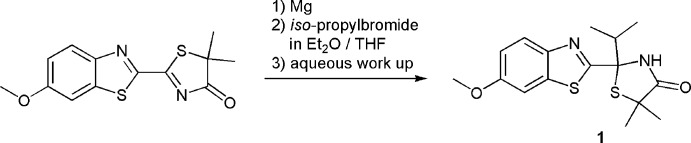
The formation of the title compound.

## supplementary crystallographic information

### 1. Comment

Luciferin, especially the class which is produced by the firefly *Photinus pyralis*, is of particular interest because of its bioluminescence and chemoluminescence properties (Naumov *et al.*, 2009). Dimethyloxyluciferin, one prominent derivative which is known for its ability to emit visible light in the red region (Branchini *et al.*, 2002), was further investigated in our group focusing on the modification on the 4-position of the thiazoline ring (Würfel, 2012). An extension of the chromophore should give rise to new dimethylluciferin derivatives with altered absorption and emission properties. The nucleophilic attack of isopropylmagnesium bromide with dimethyloxyluciferin should lead to a tertiary alcohol at the 4-position of the thiazoline ring. Subsequent dehydration reaction should form a 2-propylene substructure, thus representing a carbon extended luciferin derivative.

However, the dimethyloxyluciferin derivative did not react in this expected manner. The strong carbon nucleophile exclusively attacked the 2-position of the thiazoline ring leading, after aqueous work-up, to a racemic mixture of R,S-thiazolidines. C8 became an *sp*^3^ carbon (C8A—C1A = 1.524 (2) Å and C8B—C1B = 1.520 (2) Å), which results in the loss of the conjugation with the benzothiazole parent moiety. The thiazolidine rings are almost coplanar, with a dihedral angle of 10.32 (4)°, due to a dimer formation of the (*R*)- and (S)- enantiomers in the asymmetric unit. The dimer structure results from two hydrogen bonds between the amide moieties of the thiazolidine rings [N(A)—H···O(B) = 2.942 (2) Å and N(B)—H···O(A) = 2.802 (2) Å] from the two symmetry-independent molecules A and B. The most noticeable difference between these two molecules is the relative orientation of the benzothiazole moiety due to rotation around the C1—C8 bond. The resulting torsion angles S1—C1—C8—S2 are -19.4 (2)° (molecule A) and 100.6 (1)° (molecule B).

### 2. Experimental

All chemicals were synthesized according to given literature or purchased from commercial sources. All solvents were purified and dried according to Armarego & Chai (2009). 215 mg (8.85 mmol) of magnesium turnings in 20 ml of dry diethylether and a catalytic amount of iodine are placed in a 100 ml two-necked round-bottomed flask. 0.9 ml (9.60 mmol) 2-bromopropane was added. After a slight exothermic reaction, the mixture was refluxed for 1 h then cooled to room temperature. To that mixture 1.73 g (5.90 mmol) of 2-(6-methoxybenzothiazol-2-yl)-5,5-dimethylthiazolin-4-one in 20 ml of dry THF was added. The mixture was refluxed for 2 h, cooled to room temperature and hydrolysed with 10 g of ice and 10 ml of saturated NH_4_Cl solution, then extracted with ethyl acetate (3 × 20 ml). The extract was dried over MgSO_4_, filtered and distilled off. The remaining solid was purified by crystallization from n-heptane/ethyl acetate, yield: 70%, 1.43 g (4.25 mmol). 2-(6-Methoxybenzothiazol-2-yl)-5,5-dimethylthiazolin-4-one was synthesized from 2-cyano-6-methoxybenzothiazole and ethyl 2-mercapto-2-methylpropanoate according to Würfel (2012).

Light-yellow single crystals were obtained by dissolving the title compound at reflux temperature in n-heptane/ethyl acetate and, after cooling to room temperature, left alone in a closed vessel for several days. Elemental analysis, calculated for C_16_H_20_N_2_O_2_S_2_: C 57.11, H 5.99, N 8.33, S 19.06%; found: C 57.25, H 6.06, N 8.46, S 19.14.

### 3. Refinement

All H atoms were located from difference Fourier maps and freely refined.

### Crystal data

**Table:**

C_16_H_20_N_2_O_2_S_2_	*V* = 1687.86 (7) Å^3^
*M**_r_* = 336.46	*Z* = 4
Triclinic, *P*1	*F*(000) = 712
*a* = 11.3755 (3) Å	*D*_x_ = 1.324 Mg m^−^^3^
*b* = 11.9028 (3) Å	Mo *K*α radiation, λ = 0.71073 Å
*c* = 12.5261 (3) Å	µ = 0.32 mm^−^^1^
α = 86.122 (1)°	*T* = 133 K
β = 85.949 (1)°	Prism, colourless
γ = 89.206 (1)°	0.06 × 0.05 × 0.05 mm

### Data collection

**Table:**

Nonius KappaCCD diffractometer	6827 reflections with *I* > 2σ(*I*)
Radiation source: fine-focus sealed tube	*R*_int_ = 0.019
Graphite monochromator	θ_max_ = 27.5°, θ_min_ = 2.8°
φ and ω scans	*h* = −14→14
10948 measured reflections	*k* = −15→15
7580 independent reflections	*l* = −16→16

### Refinement

**Table:**

Refinement on *F*^2^	Primary atom site location: structure-invariant direct methods
Least-squares matrix: full	Secondary atom site location: difference Fourier map
*R*[*F*^2^ > 2σ(*F*^2^)] = 0.039	Hydrogen site location: difference Fourier map
*wR*(*F*^2^) = 0.088	All H-atom parameters refined
*S* = 1.08	*w* = 1/[σ^2^(*F*_o_^2^) + (0.0132*P*)^2^ + 1.7538*P*] where *P* = (*F*_o_^2^ + 2*F*_c_^2^)/3
7580 reflections	(Δ/σ)_max_ = 0.001
557 parameters	Δρ_max_ = 0.37 e Å^−^^3^
0 restraints	Δρ_min_ = −0.28 e Å^−^^3^

### Special details

**Table:**

Geometry. All e.s.d.'s (except the e.s.d. in the dihedral angle between two l.s. planes) are estimated using the full covariance matrix. The cell e.s.d.'s are taken into account individually in the estimation of e.s.d.'s in distances, angles and torsion angles; correlations between e.s.d.'s in cell parameters are only used when they are defined by crystal symmetry. An approximate (isotropic) treatment of cell e.s.d.'s is used for estimating e.s.d.'s involving l.s. planes.
Refinement. Refinement of *F*^2^ against ALL reflections. The weighted *R*-factor *wR* and goodness of fit *S* are based on *F*^2^, conventional *R*-factors *R* are based on *F*, with *F* set to zero for negative *F*^2^. The threshold expression of *F*^2^ > σ(*F*^2^) is used only for calculating *R*-factors(gt) *etc*. and is not relevant to the choice of reflections for refinement. *R*-factors based on *F*^2^ are statistically about twice as large as those based on *F*, and *R*-factors based on ALL data will be even larger.

### Fractional atomic coordinates and isotropic or equivalent isotropic displacement parameters (Å^2^)

**Table:**

	*x*	*y*	*z*	*U*_iso_*/*U*_eq_	
S1A	0.28415 (4)	0.06581 (4)	0.60425 (3)	0.02114 (10)	
S2A	0.45070 (4)	0.03006 (3)	0.78944 (3)	0.02050 (10)	
O1A	−0.05418 (13)	0.14410 (12)	0.34536 (12)	0.0325 (3)	
O2A	0.61551 (12)	0.30544 (11)	0.70129 (13)	0.0343 (3)	
N1A	0.16890 (13)	0.21511 (12)	0.71336 (12)	0.0214 (3)	
N2A	0.43175 (13)	0.24918 (12)	0.76326 (12)	0.0195 (3)	
H1NA	0.406 (2)	0.313 (2)	0.762 (2)	0.040 (7)*	
C1A	0.26141 (16)	0.15101 (14)	0.71262 (13)	0.0189 (3)	
C2A	0.15831 (15)	0.12492 (14)	0.55230 (14)	0.0203 (3)	
C3A	0.10820 (17)	0.10210 (15)	0.45770 (15)	0.0241 (4)	
H3A	0.1418 (18)	0.0513 (17)	0.4144 (16)	0.020 (5)*	
C4A	0.00488 (17)	0.15792 (15)	0.43471 (15)	0.0257 (4)	
C5A	−0.04879 (18)	0.23308 (17)	0.50568 (17)	0.0301 (4)	
H5A	−0.123 (2)	0.269 (2)	0.489 (2)	0.043 (7)*	
C6A	0.00094 (18)	0.25527 (16)	0.59920 (17)	0.0281 (4)	
H6A	−0.038 (2)	0.306 (2)	0.649 (2)	0.041 (7)*	
C7A	0.10746 (16)	0.20125 (15)	0.62280 (14)	0.0212 (3)	
C8A	0.35339 (15)	0.15453 (14)	0.79510 (13)	0.0188 (3)	
C9A	0.54381 (16)	0.23130 (15)	0.73098 (15)	0.0227 (4)	
C10A	0.58045 (16)	0.10721 (14)	0.73250 (15)	0.0216 (3)	
C11A	−0.0003 (2)	0.07124 (18)	0.27038 (17)	0.0338 (5)	
H11C	0.010 (2)	−0.006 (2)	0.3025 (19)	0.035 (6)*	
H11B	−0.055 (2)	0.0699 (19)	0.2129 (19)	0.036 (6)*	
H11A	0.076 (2)	0.101 (2)	0.2387 (19)	0.034 (6)*	
C12A	0.68389 (19)	0.08635 (19)	0.8030 (2)	0.0349 (5)	
H12C	0.750 (2)	0.131 (2)	0.773 (2)	0.040 (7)*	
H12B	0.660 (2)	0.106 (2)	0.877 (2)	0.042 (7)*	
H12A	0.704 (2)	0.006 (2)	0.804 (2)	0.042 (7)*	
C13A	0.6148 (2)	0.07723 (17)	0.61714 (17)	0.0301 (4)	
H13C	0.684 (2)	0.125 (2)	0.5885 (19)	0.040 (7)*	
H13A	0.551 (2)	0.094 (2)	0.5703 (19)	0.035 (6)*	
H13B	0.633 (2)	−0.002 (2)	0.6156 (19)	0.037 (6)*	
C14A	0.29315 (17)	0.16514 (15)	0.90837 (14)	0.0223 (4)	
H14A	0.2439 (19)	0.2328 (18)	0.9028 (17)	0.026 (5)*	
C15A	0.3822 (2)	0.18177 (19)	0.99156 (16)	0.0297 (4)	
H15C	0.433 (2)	0.117 (2)	0.9995 (18)	0.033 (6)*	
H15B	0.431 (2)	0.249 (2)	0.9696 (19)	0.039 (7)*	
H15A	0.342 (2)	0.194 (2)	1.062 (2)	0.038 (6)*	
C16A	0.2143 (2)	0.06453 (18)	0.94200 (16)	0.0307 (4)	
H16C	0.177 (2)	0.0724 (19)	1.0127 (19)	0.032 (6)*	
H16B	0.149 (2)	0.059 (2)	0.893 (2)	0.050 (7)*	
H16A	0.260 (2)	−0.005 (2)	0.944 (2)	0.043 (7)*	
S1B	0.68977 (4)	0.53085 (4)	0.91211 (3)	0.02025 (10)	
S2B	0.57063 (4)	0.75544 (3)	0.70861 (4)	0.02149 (10)	
O1B	0.99279 (11)	0.56823 (11)	1.20270 (10)	0.0247 (3)	
O2B	0.37416 (11)	0.49130 (10)	0.75042 (10)	0.0225 (3)	
N1B	0.82852 (13)	0.67362 (12)	0.80061 (12)	0.0206 (3)	
N2B	0.56709 (13)	0.53534 (12)	0.71793 (11)	0.0173 (3)	
H1NB	0.5860 (18)	0.4740 (19)	0.7118 (16)	0.019 (5)*	
C1B	0.73372 (15)	0.61588 (14)	0.79671 (13)	0.0180 (3)	
C2B	0.81058 (15)	0.57803 (14)	0.97259 (14)	0.0195 (3)	
C3B	0.84486 (16)	0.54766 (15)	1.07565 (14)	0.0206 (3)	
H3B	0.7977 (18)	0.4988 (18)	1.1222 (17)	0.023 (5)*	
C4B	0.94756 (15)	0.59370 (15)	1.10563 (14)	0.0209 (3)	
C5B	1.01291 (16)	0.66994 (15)	1.03522 (15)	0.0239 (4)	
H5B	1.083 (2)	0.7007 (18)	1.0588 (17)	0.028 (6)*	
C6B	0.97773 (16)	0.69941 (16)	0.93396 (15)	0.0243 (4)	
H6B	1.0225 (18)	0.7450 (17)	0.8859 (16)	0.020 (5)*	
C7B	0.87480 (15)	0.65293 (14)	0.90076 (14)	0.0191 (3)	
C8B	0.65620 (15)	0.62225 (13)	0.70244 (13)	0.0173 (3)	
C9B	0.42712 (16)	0.68758 (14)	0.73455 (15)	0.0226 (4)	
C10B	0.45328 (15)	0.56124 (14)	0.73509 (13)	0.0183 (3)	
C11B	0.92381 (18)	0.49480 (19)	1.27642 (16)	0.0289 (4)	
H11F	0.965 (2)	0.4887 (19)	1.3430 (19)	0.034 (6)*	
H11E	0.920 (2)	0.420 (2)	1.2484 (19)	0.034 (6)*	
H11D	0.843 (2)	0.528 (2)	1.2912 (19)	0.039 (6)*	
C12B	0.3464 (2)	0.72068 (19)	0.6450 (2)	0.0376 (5)	
H12F	0.272 (2)	0.682 (2)	0.661 (2)	0.047 (7)*	
H12E	0.329 (2)	0.801 (2)	0.644 (2)	0.044 (7)*	
H12D	0.384 (2)	0.701 (2)	0.573 (2)	0.046 (7)*	
C13B	0.3702 (2)	0.71541 (18)	0.8440 (2)	0.0364 (5)	
H13F	0.295 (2)	0.681 (2)	0.8564 (19)	0.039 (7)*	
H13E	0.422 (2)	0.687 (2)	0.903 (2)	0.046 (7)*	
H13D	0.359 (2)	0.795 (2)	0.844 (2)	0.050 (7)*	
C14B	0.73008 (16)	0.61496 (15)	0.59462 (14)	0.0205 (3)	
H14B	0.7845 (18)	0.6783 (17)	0.5920 (16)	0.021 (5)*	
C15B	0.65467 (19)	0.62882 (18)	0.49827 (16)	0.0279 (4)	
H15F	0.593 (2)	0.569 (2)	0.5022 (18)	0.035 (6)*	
H15E	0.615 (2)	0.702 (2)	0.4936 (19)	0.035 (6)*	
H15D	0.704 (2)	0.624 (2)	0.434 (2)	0.038 (6)*	
C16B	0.79781 (18)	0.50310 (17)	0.59283 (16)	0.0261 (4)	
H16F	0.746 (2)	0.4405 (19)	0.5836 (17)	0.029 (6)*	
H16E	0.837 (2)	0.4845 (19)	0.6607 (19)	0.033 (6)*	
H16D	0.859 (2)	0.508 (2)	0.534 (2)	0.038 (6)*	

### Atomic displacement parameters (Å^2^)

**Table:**

	*U*^11^	*U*^22^	*U*^33^	*U*^12^	*U*^13^	*U*^23^
S1A	0.0240 (2)	0.0204 (2)	0.0200 (2)	0.00335 (16)	−0.00505 (16)	−0.00566 (16)
S2A	0.0250 (2)	0.01400 (19)	0.0225 (2)	0.00315 (16)	−0.00349 (16)	−0.00055 (15)
O1A	0.0353 (8)	0.0318 (7)	0.0329 (8)	−0.0037 (6)	−0.0180 (6)	−0.0034 (6)
O2A	0.0282 (7)	0.0191 (7)	0.0545 (10)	−0.0009 (5)	0.0045 (7)	−0.0028 (6)
N1A	0.0248 (8)	0.0202 (7)	0.0195 (7)	0.0025 (6)	−0.0038 (6)	−0.0021 (6)
N2A	0.0229 (7)	0.0121 (7)	0.0237 (8)	0.0027 (6)	−0.0033 (6)	−0.0015 (6)
C1A	0.0262 (9)	0.0146 (7)	0.0161 (8)	−0.0006 (6)	−0.0022 (6)	−0.0005 (6)
C2A	0.0220 (8)	0.0180 (8)	0.0210 (8)	−0.0013 (6)	−0.0031 (7)	0.0006 (6)
C3A	0.0290 (9)	0.0201 (8)	0.0238 (9)	−0.0032 (7)	−0.0055 (7)	−0.0031 (7)
C4A	0.0293 (10)	0.0221 (9)	0.0266 (9)	−0.0058 (7)	−0.0104 (8)	0.0007 (7)
C5A	0.0262 (10)	0.0258 (9)	0.0396 (11)	0.0020 (8)	−0.0124 (8)	−0.0016 (8)
C6A	0.0274 (10)	0.0247 (9)	0.0334 (10)	0.0049 (7)	−0.0081 (8)	−0.0061 (8)
C7A	0.0224 (8)	0.0197 (8)	0.0218 (8)	0.0005 (7)	−0.0045 (7)	−0.0008 (7)
C8A	0.0239 (8)	0.0143 (7)	0.0186 (8)	0.0020 (6)	−0.0039 (7)	−0.0023 (6)
C9A	0.0262 (9)	0.0173 (8)	0.0251 (9)	0.0013 (7)	−0.0042 (7)	−0.0018 (7)
C10A	0.0219 (8)	0.0168 (8)	0.0260 (9)	0.0028 (6)	−0.0020 (7)	−0.0009 (7)
C11A	0.0453 (13)	0.0294 (11)	0.0286 (10)	−0.0082 (9)	−0.0140 (9)	−0.0025 (8)
C12A	0.0274 (10)	0.0323 (11)	0.0456 (13)	0.0035 (9)	−0.0117 (9)	0.0026 (10)
C13A	0.0362 (11)	0.0226 (9)	0.0306 (10)	0.0021 (8)	0.0054 (9)	−0.0031 (8)
C14A	0.0285 (9)	0.0222 (9)	0.0168 (8)	0.0050 (7)	−0.0031 (7)	−0.0040 (7)
C15A	0.0369 (11)	0.0333 (11)	0.0205 (9)	0.0062 (9)	−0.0077 (8)	−0.0082 (8)
C16A	0.0379 (11)	0.0327 (11)	0.0208 (9)	−0.0032 (9)	0.0011 (8)	0.0000 (8)
S1B	0.0208 (2)	0.0223 (2)	0.0177 (2)	−0.00328 (16)	−0.00279 (16)	0.00074 (16)
S2B	0.0231 (2)	0.01373 (19)	0.0278 (2)	−0.00026 (16)	−0.00344 (17)	−0.00067 (16)
O1B	0.0224 (6)	0.0331 (7)	0.0195 (6)	0.0026 (5)	−0.0057 (5)	−0.0036 (5)
O2B	0.0198 (6)	0.0185 (6)	0.0294 (7)	−0.0011 (5)	−0.0019 (5)	−0.0028 (5)
N1B	0.0206 (7)	0.0194 (7)	0.0222 (7)	−0.0011 (6)	−0.0041 (6)	−0.0018 (6)
N2B	0.0210 (7)	0.0107 (7)	0.0202 (7)	−0.0003 (5)	−0.0014 (6)	−0.0015 (5)
C1B	0.0199 (8)	0.0170 (8)	0.0169 (8)	0.0014 (6)	−0.0003 (6)	−0.0013 (6)
C2B	0.0202 (8)	0.0187 (8)	0.0200 (8)	0.0018 (6)	−0.0013 (6)	−0.0046 (6)
C3B	0.0214 (8)	0.0229 (8)	0.0177 (8)	0.0020 (7)	−0.0009 (7)	−0.0032 (7)
C4B	0.0213 (8)	0.0234 (8)	0.0187 (8)	0.0063 (7)	−0.0029 (7)	−0.0058 (7)
C5B	0.0222 (9)	0.0232 (9)	0.0276 (9)	−0.0007 (7)	−0.0065 (7)	−0.0047 (7)
C6B	0.0227 (9)	0.0233 (9)	0.0267 (9)	−0.0037 (7)	−0.0029 (7)	0.0008 (7)
C7B	0.0199 (8)	0.0183 (8)	0.0194 (8)	0.0020 (6)	−0.0024 (6)	−0.0036 (6)
C8B	0.0192 (8)	0.0147 (7)	0.0182 (8)	−0.0011 (6)	−0.0031 (6)	−0.0006 (6)
C9B	0.0214 (8)	0.0159 (8)	0.0308 (10)	0.0014 (6)	−0.0044 (7)	−0.0010 (7)
C10B	0.0218 (8)	0.0167 (8)	0.0168 (8)	−0.0004 (6)	−0.0036 (6)	−0.0027 (6)
C11B	0.0233 (9)	0.0436 (12)	0.0194 (9)	0.0020 (8)	−0.0027 (7)	0.0003 (8)
C12B	0.0299 (11)	0.0242 (10)	0.0591 (15)	0.0004 (8)	−0.0195 (10)	0.0114 (10)
C13B	0.0382 (12)	0.0220 (10)	0.0478 (14)	−0.0031 (9)	0.0157 (10)	−0.0122 (9)
C14B	0.0215 (8)	0.0215 (8)	0.0187 (8)	−0.0050 (7)	−0.0010 (7)	−0.0009 (6)
C15B	0.0321 (10)	0.0329 (11)	0.0189 (9)	−0.0026 (8)	−0.0043 (8)	−0.0002 (8)
C16B	0.0259 (9)	0.0276 (10)	0.0245 (9)	0.0017 (8)	0.0028 (8)	−0.0049 (8)

### Geometric parameters (Å, º)

**Table:**

S1A—C2A	1.7347 (18)	S1B—C2B	1.7331 (18)
S1A—C1A	1.7513 (17)	S1B—C1B	1.7544 (17)
S2A—C10A	1.8250 (18)	S2B—C9B	1.8301 (18)
S2A—C8A	1.8393 (17)	S2B—C8B	1.8521 (17)
O1A—C4A	1.366 (2)	O1B—C4B	1.367 (2)
O1A—C11A	1.423 (3)	O1B—C11B	1.428 (2)
O2A—C9A	1.231 (2)	O2B—C10B	1.231 (2)
N1A—C1A	1.291 (2)	N1B—C1B	1.292 (2)
N1A—C7A	1.394 (2)	N1B—C7B	1.400 (2)
N2A—C9A	1.329 (2)	N2B—C10B	1.333 (2)
N2A—C8A	1.463 (2)	N2B—C8B	1.452 (2)
N2A—H1NA	0.81 (3)	N2B—H1NB	0.77 (2)
C1A—C8A	1.524 (2)	C1B—C8B	1.520 (2)
C2A—C3A	1.395 (2)	C2B—C3B	1.398 (2)
C2A—C7A	1.401 (2)	C2B—C7B	1.398 (2)
C3A—C4A	1.381 (3)	C3B—C4B	1.383 (2)
C3A—H3A	0.90 (2)	C3B—H3B	0.94 (2)
C4A—C5A	1.407 (3)	C4B—C5B	1.404 (3)
C5A—C6A	1.379 (3)	C5B—C6B	1.378 (3)
C5A—H5A	0.97 (3)	C5B—H5B	0.95 (2)
C6A—C7A	1.404 (3)	C6B—C7B	1.403 (2)
C6A—H6A	0.98 (3)	C6B—H6B	0.91 (2)
C8A—C14A	1.544 (2)	C8B—C14B	1.546 (2)
C9A—C10A	1.528 (2)	C9B—C12B	1.527 (3)
C10A—C12A	1.528 (3)	C9B—C10B	1.529 (2)
C10A—C13A	1.534 (3)	C9B—C13B	1.529 (3)
C11A—H11C	0.98 (2)	C11B—H11F	0.99 (2)
C11A—H11B	0.98 (2)	C11B—H11E	0.99 (2)
C11A—H11A	0.99 (2)	C11B—H11D	1.00 (3)
C12A—H12C	0.96 (3)	C12B—H12F	0.97 (3)
C12A—H12B	0.99 (3)	C12B—H12E	0.97 (3)
C12A—H12A	0.98 (3)	C12B—H12D	1.01 (3)
C13A—H13C	1.01 (2)	C13B—H13F	0.96 (3)
C13A—H13A	0.98 (2)	C13B—H13E	1.01 (3)
C13A—H13B	0.96 (2)	C13B—H13D	0.95 (3)
C14A—C16A	1.525 (3)	C14B—C15B	1.528 (3)
C14A—C15A	1.528 (3)	C14B—C16B	1.530 (3)
C14A—H14A	0.98 (2)	C14B—H14B	0.98 (2)
C15A—H15C	0.96 (2)	C15B—H15F	1.00 (2)
C15A—H15B	1.00 (3)	C15B—H15E	0.97 (2)
C15A—H15A	0.98 (3)	C15B—H15D	0.95 (3)
C16A—H16C	0.96 (2)	C16B—H16F	0.97 (2)
C16A—H16B	1.00 (3)	C16B—H16E	1.00 (2)
C16A—H16A	0.97 (3)	C16B—H16D	0.98 (2)
			
C2A—S1A—C1A	88.64 (8)	C2B—S1B—C1B	88.74 (8)
C10A—S2A—C8A	95.33 (8)	C9B—S2B—C8B	95.21 (8)
C4A—O1A—C11A	116.31 (16)	C4B—O1B—C11B	116.02 (14)
C1A—N1A—C7A	110.12 (15)	C1B—N1B—C7B	109.91 (15)
C9A—N2A—C8A	120.58 (14)	C10B—N2B—C8B	121.29 (14)
C9A—N2A—H1NA	120.0 (18)	C10B—N2B—H1NB	119.9 (16)
C8A—N2A—H1NA	119.4 (18)	C8B—N2B—H1NB	118.7 (16)
N1A—C1A—C8A	122.99 (15)	N1B—C1B—C8B	124.46 (15)
N1A—C1A—S1A	116.55 (13)	N1B—C1B—S1B	116.50 (13)
C8A—C1A—S1A	120.26 (12)	C8B—C1B—S1B	118.95 (12)
C3A—C2A—C7A	122.32 (17)	C3B—C2B—C7B	122.71 (16)
C3A—C2A—S1A	128.22 (14)	C3B—C2B—S1B	127.77 (14)
C7A—C2A—S1A	109.43 (13)	C7B—C2B—S1B	109.50 (13)
C4A—C3A—C2A	117.58 (18)	C4B—C3B—C2B	117.57 (17)
C4A—C3A—H3A	121.8 (13)	C4B—C3B—H3B	122.5 (13)
C2A—C3A—H3A	120.6 (13)	C2B—C3B—H3B	119.9 (13)
O1A—C4A—C3A	124.15 (18)	O1B—C4B—C3B	123.43 (16)
O1A—C4A—C5A	114.95 (17)	O1B—C4B—C5B	115.85 (16)
C3A—C4A—C5A	120.89 (17)	C3B—C4B—C5B	120.72 (16)
C6A—C5A—C4A	121.33 (18)	C6B—C5B—C4B	121.03 (17)
C6A—C5A—H5A	119.7 (15)	C6B—C5B—H5B	120.5 (13)
C4A—C5A—H5A	119.0 (15)	C4B—C5B—H5B	118.5 (13)
C5A—C6A—C7A	118.62 (18)	C5B—C6B—C7B	119.55 (17)
C5A—C6A—H6A	120.5 (15)	C5B—C6B—H6B	121.6 (13)
C7A—C6A—H6A	120.9 (15)	C7B—C6B—H6B	118.7 (13)
N1A—C7A—C2A	115.21 (15)	C2B—C7B—N1B	115.35 (15)
N1A—C7A—C6A	125.55 (17)	C2B—C7B—C6B	118.41 (16)
C2A—C7A—C6A	119.23 (16)	N1B—C7B—C6B	126.25 (16)
N2A—C8A—C1A	108.24 (13)	N2B—C8B—C1B	109.97 (13)
N2A—C8A—C14A	111.57 (14)	N2B—C8B—C14B	111.78 (13)
C1A—C8A—C14A	110.48 (14)	C1B—C8B—C14B	111.54 (14)
N2A—C8A—S2A	104.11 (11)	N2B—C8B—S2B	103.99 (11)
C1A—C8A—S2A	110.32 (11)	C1B—C8B—S2B	106.87 (11)
C14A—C8A—S2A	111.89 (12)	C14B—C8B—S2B	112.33 (11)
O2A—C9A—N2A	125.08 (17)	C12B—C9B—C10B	109.06 (15)
O2A—C9A—C10A	120.46 (17)	C12B—C9B—C13B	111.26 (19)
N2A—C9A—C10A	114.46 (15)	C10B—C9B—C13B	109.43 (15)
C12A—C10A—C9A	109.71 (16)	C12B—C9B—S2B	110.87 (14)
C12A—C10A—C13A	110.66 (17)	C10B—C9B—S2B	105.22 (12)
C9A—C10A—C13A	108.76 (15)	C13B—C9B—S2B	110.80 (14)
C12A—C10A—S2A	110.76 (14)	O2B—C10B—N2B	124.19 (16)
C9A—C10A—S2A	105.09 (12)	O2B—C10B—C9B	121.55 (16)
C13A—C10A—S2A	111.68 (13)	N2B—C10B—C9B	114.26 (14)
O1A—C11A—H11C	111.9 (14)	O1B—C11B—H11F	106.0 (13)
O1A—C11A—H11B	105.3 (14)	O1B—C11B—H11E	110.1 (14)
H11C—C11A—H11B	109.3 (19)	H11F—C11B—H11E	109.2 (19)
O1A—C11A—H11A	111.7 (14)	O1B—C11B—H11D	110.5 (14)
H11C—C11A—H11A	110.0 (19)	H11F—C11B—H11D	109.6 (19)
H11B—C11A—H11A	108.4 (19)	H11E—C11B—H11D	111.3 (19)
C10A—C12A—H12C	109.0 (15)	C9B—C12B—H12F	108.6 (16)
C10A—C12A—H12B	109.2 (15)	C9B—C12B—H12E	109.7 (15)
H12C—C12A—H12B	111 (2)	H12F—C12B—H12E	107 (2)
C10A—C12A—H12A	108.0 (15)	C9B—C12B—H12D	110.6 (15)
H12C—C12A—H12A	111 (2)	H12F—C12B—H12D	111 (2)
H12B—C12A—H12A	109 (2)	H12E—C12B—H12D	110 (2)
C10A—C13A—H13C	108.3 (14)	C9B—C13B—H13F	110.3 (15)
C10A—C13A—H13A	111.6 (14)	C9B—C13B—H13E	109.8 (15)
H13C—C13A—H13A	107.6 (19)	H13F—C13B—H13E	109 (2)
C10A—C13A—H13B	110.1 (14)	C9B—C13B—H13D	108.6 (16)
H13C—C13A—H13B	112 (2)	H13F—C13B—H13D	108 (2)
H13A—C13A—H13B	107.3 (19)	H13E—C13B—H13D	111 (2)
C16A—C14A—C15A	111.16 (16)	C15B—C14B—C16B	109.80 (15)
C16A—C14A—C8A	111.16 (15)	C15B—C14B—C8B	112.39 (15)
C15A—C14A—C8A	112.13 (16)	C16B—C14B—C8B	110.38 (14)
C16A—C14A—H14A	108.6 (13)	C15B—C14B—H14B	109.0 (12)
C15A—C14A—H14A	108.0 (13)	C16B—C14B—H14B	110.8 (12)
C8A—C14A—H14A	105.5 (13)	C8B—C14B—H14B	104.4 (12)
C14A—C15A—H15C	110.8 (14)	C14B—C15B—H15F	110.9 (13)
C14A—C15A—H15B	110.2 (14)	C14B—C15B—H15E	112.0 (14)
H15C—C15A—H15B	109 (2)	H15F—C15B—H15E	108.2 (19)
C14A—C15A—H15A	111.0 (14)	C14B—C15B—H15D	109.5 (15)
H15C—C15A—H15A	108.0 (19)	H15F—C15B—H15D	109.4 (19)
H15B—C15A—H15A	107.7 (19)	H15E—C15B—H15D	107 (2)
C14A—C16A—H16C	110.1 (13)	C14B—C16B—H16F	111.8 (13)
C14A—C16A—H16B	111.6 (15)	C14B—C16B—H16E	112.2 (13)
H16C—C16A—H16B	107 (2)	H16F—C16B—H16E	106.6 (18)
C14A—C16A—H16A	110.7 (15)	C14B—C16B—H16D	109.0 (14)
H16C—C16A—H16A	108 (2)	H16F—C16B—H16D	109.1 (19)
H16B—C16A—H16A	110 (2)	H16E—C16B—H16D	108.0 (19)
			
S1A—C1A—C8A—S2A	−19.39 (17)	S1B—C1B—C8B—S2B	100.58 (12)

### Hydrogen-bond geometry (Å, º)

**Table:**

*D*—H···*A*	*D*—H	H···*A*	*D*···*A*	*D*—H···*A*
N2*A*—H1*NA*···O2*B*	0.81 (3)	2.15 (3)	2.9429 (19)	168 (2)
N2*B*—H1*NB*···O2*A*	0.77 (2)	2.04 (2)	2.802 (2)	173 (2)
